# Seroprevalence of *Toxoplasma gondii* in wild boars (*Sus scrofa*) hunted in Ukraine

**DOI:** 10.1016/j.ijppaw.2023.100901

**Published:** 2023-12-29

**Authors:** Maryna Galat, Gaston Moré, Caroline F. Frey, Ganna Kovalenko, Inna Maliuk, Ihor Halka, Mykola Sytiuk, Maksym Bezymennyi, Vladyslav Galat, Pikka Jokelainen

**Affiliations:** a Institute of Parasitology, Department of Infectious Diseases and Pathobiology, Vetsuisse Faculty University of Bern, Länggassstrasse 122, 3012, Bern, Switzerland; b Faculty of Veterinary Medicine, National University of Life and Environmental Sciences of Ukraine, Heroiv Oborony Str. 15, 03041, Kyiv, Ukraine; c Institute of Veterinary Medicine of the National Academy of Agrarian Sciences of Ukraine, Donetska Str. 30, 03151, Kyiv, Ukraine; d Division of Virology, Department of Pathology, University of Cambridge, Cambridge, UK; e Infectious Disease Preparedness, Statens Serum Institut, Artillerivej 5, 2300, Copenhagen S, Denmark; f Faculty of Veterinary Medicine, University of Helsinki, P.O. Box 66, 00014, Helsinki, Finland; g Department of Biological Sciences, University of Alaska Anchorage, Anchorage, AK, USA; h Institute of Veterinary Medicine and Animal Sciences, Estonian University of Life Sciences, Kreutzwaldi 62, 51006, Tartu, Estonia

**Keywords:** Antibodies, Serology, Toxoplasmosis, Ukraine, Wild boars, Zoonosis

## Abstract

*Toxoplasma gondii* is an important zoonotic parasite worldwide, but it has received limited attention in Ukraine. A seroepidemiological study was conducted and samples from 452 wild boars that had been hunted in 2006–2011 in 23 of the 25 regions of Ukraine were tested to estimate *T*. *gondii* seroprevalence. A locally available commercial enzyme-linked immunosorbent assay (ELISA) was used for the investigation. Additionally, we tested 92 of the sera using a widely used commercial multi-species ELISA and an indirect immunofluorescence antibody test (IFAT). With the locally available ELISA, 35 of the 452 wild boars tested positive, yielding a seroprevalence estimate of 7.7% (95% confidence interval 5.5–10.5). The seropositive wild boars originated from eight of the regions. Using the majority criteria, 10/92 samples tested using both ELISAs and the IFAT were considered positive, yielding an estimated seroprevalence of 10.9% within the subset of samples. The highest seroprevalence was observed in wild boars hunted in Luhans'k (30.0%), Odesa (17.7%) and Kharkiv (12.7%). Seroprevalence was higher in older animals (13.3% for age group >12 months and 7.7% for age group ≤12 months). This is the first seroepidemiological study of *T. gondii* in wild boars in Ukraine. Assuming that seropositivity indicates presence of infectious parasites in the tissues, eating undercooked meat of wild boars hunted in Ukraine could be a potential source of infection to other hosts, including humans.

## Highlights

• This is the first seroepidemiological study of *T. gondii* in wild boars in Ukraine.
• Sera of wild boars from Ukraine were tested with three serological methods.
• Overall *Toxoplasma gondii* seroprevalence estimate was 7.7%.

## Graphical abstract

**Figure:**
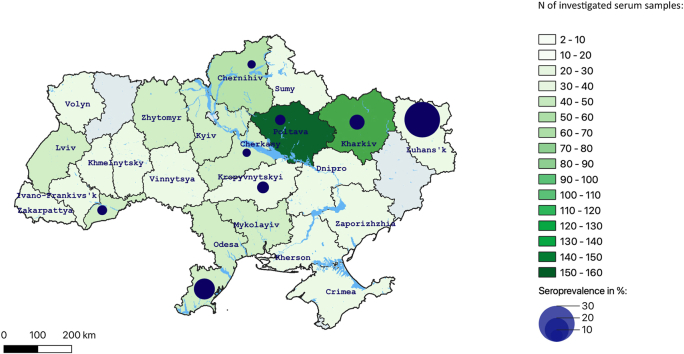

## 1 Introduction

Toxoplasmosis as a zoonotic disease caused by the protozoan parasite *Toxoplasma gondii* (Dubey, 2010). Little information about *T. gondii* has been available from Ukraine. The country is divided into nine major basins by major watersheds: the Dnipro River Basin, Dniester River Basin, Danube River Basin, Vistula (Western Bug) River Basin, Southern Bug River Basin, Don (Siverskyi Donets) River Basin, Azov Sea Rivers Basin, Black Sea Rivers Basin and Crimea Rivers Basin.

Wild boars (*Sus scrofa*, Linnaeus, 1758) are a popular game species in Ukraine. The number of wild boars in Ukraine has been estimated as 44,800 in 2006, 65,000 in 2011, and 40,700 in 2017; the average hunting bag was 5900 wild boars in 2006–2014 and 20,000 wild boars in 2015–2016 (Hoetskуу et al., 2017). There has been a decrease in the wild boar population since 2015, related to a program to reduce the spread of African swine fever in the country. Very recent data on population size are not available.

Wild boars are considered a good indicator host species for presence and spread of *T. gondii*, and they can also constitute a source of infection to other hosts (Gauss et al., 2005; Richomme et al., 2009; Bassi et al., 2021; Papatsiros et al., 2021; Dámek et al., 2023). Various methods have been used worldwide to determine *T. gondii* seroprevalence in wild boars. Among the most common are enzyme-linked immunosorbent assays (ELISA), modified direct agglutination test, latex agglutination method, indirect immunofluorescent antibody test (IFAT), and others (Rostami et al., 2017; Cubas-Atienzar et al., 2019; Wallander et al., 2015).

The aims of our investigation were to estimate *T. gondii* seroprevalence among wild boars hunted in Ukraine and to compare the results obtained by three serological tests: locally available ELISA, commercial ELISA and IFAT.

## 2 Materials and methods

### 2.1 Ethical considerations

No wild boars were hunted for the purpose of this study. We used surplus serum samples. We did not handle personal information of the hunters.

### 2.2 Samples

The sampling frame consisted of 6840 surplus serum samples that had been collected from wild boars across Ukraine for a nationwide monitoring program of viral diseases, including African swine fever (ASF), for which all the samples tested negative (Nevolko et al., 2015; Sytiuk, 2015). The blood samples had been originally collected by hunters into syringes after the wild boars had been shot, and the hunters had provided the background information for each animal (hunting location, sex of the animal, age group of the animal based on its phenotype, and weight estimate). The sera were provided for this study by the Institute of Veterinary Medicine (Kyiv, Ukraine), which received them from the State Scientific and Institutional Institute of Laboratory Diagnostics and Veterinary Sanitary Expertise (Kyiv, Ukraine). The samples were stored frozen at −20° Celcius until and between the analyses. The samples had been collected in a time period of five years covering six subsequent hunting seasons (from 2005 to 2006 to 2010–2011).

The sample size for this study was arrived at by a calculation using OpenEpi software (Dean et al., 2013). For the calculation, the pooled seroprevalence estimates from two recent systematic reviews and meta-analyses – a global estimate of 23.0% (Rostami et al., 2017) and an estimate from Nordic-Baltic region of 33.1% (Olsen et al., 2019) – served as the expected seroprevalences and 65,000 wild boars as the population size (Hoetskуу et al., 2017), precision was set at 5% and confidence level at 95%. The minimum sample size for estimating seroprevalence in Ukraine was 271–339 wild boars. Selection of samples for this study was done categorized by region and based on availability of sufficient amount of serum. The overall number of samples included was 452. The selection and serological analyses were done blinded in regards to other background information about the samples.

The included samples had been collected in 2006 and 2008–2011. The samples originated from 23 out of 25 regions of Ukraine, all except Donets'k and Rivne. According to hunting locations, the animals were grouped in relation to the major basins as follows: River Basin group 1 (n = 98): Vistula (Western Bug) River Basin, Dniester River Basin, Danube River Basin, Southern Bug River Basin and western part of Black Sea Rivers Basin; River Basin group 2 (n = 264): Dnipro River Basin; River Basin group 3 (n = 90): Don (Siverskyi Donets) River Basin, Azov Sea Rivers Basin and Crimea Rivers Basin). Information about age group was available for 159, information about sex for 172, and information about weight group for 136 of the wild boars.

### 2.3 Serology

The first stage of this research was conducted at the laboratory of the Institute of Veterinary Medicine, the National Academy of Agrarian Sciences of Ukraine, Kyiv, Ukraine, in 2018. Altogether 360 of the sera were tested for presence of total antibodies against *T. gondii* using a locally available ELISA (VectoToxo-antibodies [*VektoTokso-antytila*], VectorBest, Novosibirsk, Russian Federation), following the instructions of the manufacturer. This kit is intended for the detection of total antibodies against *T. gondii* in human or animal serum or plasma, however it does not mention wild boars specifically. The kit was sold in Ukraine until February 24, 2022, for testing human samples only. The kit does not provide details about the antigen it is based on. A horseradish peroxidase universal conjugate is used as secondary antibody. Optical density was measured at 450 nm using an iMark Microplate Absorbance Reader (BIO-RAD). The cut-off optical density was obtained by adding 0.3 to the arithmetic mean optical density of the negative controls on the same plate. The samples with optical density equal to or higher than the cut-off optical density were considered positive, and those with lower optical density were considered negative.

Additional serological analyses of 92 of the samples were carried out at the Institute of Parasitology of the Vetsuisse Faculty (University of Bern, Bern, Switzerland), in 2023. The 92 sera were tested for *T. gondii*-specific antibodies, in addition to using the locally available ELISA, with a widely used commercial ELISA (ID-Screen Toxoplasmosis Indirect Multi-Species, ID. vet Innovative Diagnostics, Grabels, France), and with an IFAT. The IFAT was performed using tachyzoites of the RH strain coated in multi-well slides which were stored at −80 °C. As positive and negative controls, pig sera which was previously tested were included in each reaction series (Basso et al., 2013; Winter et al., 2019), as well as buffer (no conjugate, no serum) and conjugate controls (all reagents, no serum). As conjugate Anti-Pig IgG (whole molecule) – FITC antibody produced in rabbit (Sigma-Aldrich, St. Louis, MO, USA) in dilution 1:100 was used. The optimal dilutions for sera and conjugate were determined by titration. As a result sera were tested at a dilution of 1:40. Reaction was observed using a fluorescent microscope (Leitz, Laborlux S) by two different operators. Interpretation of IFAT results was done as described previously for other protozoans (Paré et al., 1995). The ELISAs were performed according to the manufacturers’ instructions. Optical density was measured using a Tecan Sunrise Reader (TECAN). Cutoff calculations followed the instructions of the manufacturers. An ELISA positivity proportion index was also calculated for each sample: optical density of the sample/mean optical density of the positive controls on the plate *100.

### 2.4 Statistical analyses

The overall seroprevalence was estimated based on results of the 452 samples. The majority criteria and confidence intervals (95% CI) for proportions (dichotomized outcome, seronegative or seropositive) were calculated using Mid-P exact of OpenEpi (Dean et al., 2013) and used to evaluate agreement between the serological tests. For visualization, a map of Ukraine was created using the QGIS software (3.28.3-Firenze).

## 3 Results

**Fig. 1:**
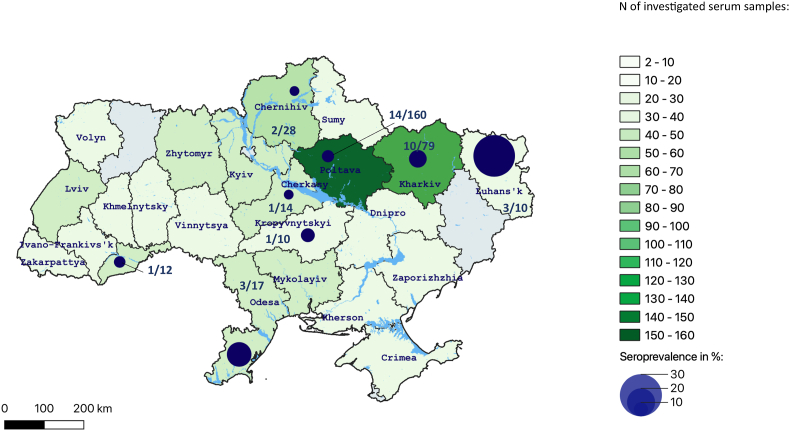
Seroprevalence of *Toxoplasma gondii* infection among wild boars by region in Ukraine, based on results from a locally available enzyme-linked immunosorbent assay (ELISA). For regions with at least one seropositive wild boar, the number of seropositive wild boards out of number of tested wild boars is shown.

With the locally available ELISA, 35 of the 452 wild boars tested positive, yielding an ELISA-seroprevalence estimate of 7.7% (95% CI 5.5–10.5). The seropositive wild boars originated from eight of the regions (Fig. 1). Table 1 shows the seroprevalence estimates by hunting season, groups of major basins of Ukraine, age group, weight group, and sex.

**Table 1:** *Toxoplasma gondii* seroprevalence in hunted wild boars (*Sus scrofa*) in Ukraine. The proportion of wild boars testing seropositive with a commercial locally available enzyme-linked immunosorbent assay (ELISA) by hunting seasons, places were wild boars were hunted according to the water basins, age groups, weight groups, and sex.

	*N*	*n* ELISA-seropositive wild boars	% ELISA-seropositive wild boars	95% confidence interval
**Hunting season**				
**Hunting seasons 2005**–**2006 and 2006**–**2007 combined**	26	4	15.4	5.1–33.1
2005–2006	7	0	0.0	0.0–34.8
2006–2007	19	4	21.1	7.1–43.3
**Hunting seasons 2007**–**2008 and 2008**–**2009 combined**	138	8	5.8	2.7–10.7
2007–2008	18	3	16.7	4.4–39.0
2008–2009	120	5	4.2	1.5–9.0
**Hunting seasons 2009**–**2010 and 2010**–**2011 combined**	288	23	8.0	5.3–11.6
2009–2010	154	6	3.9	1.6–7.9
2010–2011	134	17	12.7	7.8–19.2
**Places where wild boars were hunted according to group of major basins**				
River Basin group 1	98	5	5.1	1.9/10.9
River Basin group 2	264	18	6.8	4.2/10.4
River Barin group 3	90	12	13.3	7.4/21.6
**Age groups**				
**Age group ≤12 months**	108	8	7.4	3.5–13.6
6–8 months	43	5	11.6	4.4–23.9
8–10 months	26	0	0.0	0.0–10.9
10–12 months	39	3	7.7	2.0–19.5
**Age group >12 months**	51	8	15.7	7.6–27.6
12–18 months	20	6	30.0	13.2–52.3
**>**24 months	31	2	6.5	1.1–19.7
**Weight groups**				
**Weight group 40**–**80 kg**	62	5	8.1	3.0–17.0
40–60 kg	16	0	0.0	0.0–17.1
60–80 kg	46	5	10.9	4.1–22.5
**Weight group 80**–**120 kg**	74	8	10.8	5.1–19.5
80–100 kg	26	1	3.8	0.2–17.5
100–120 kg	48	7	14.6	6.6–26.7
**Sex**				
**Female**	11	1	9.1	0.5–37.3
**Male**	161	15	9.3	5.5–14.6

**Fig. 2:**
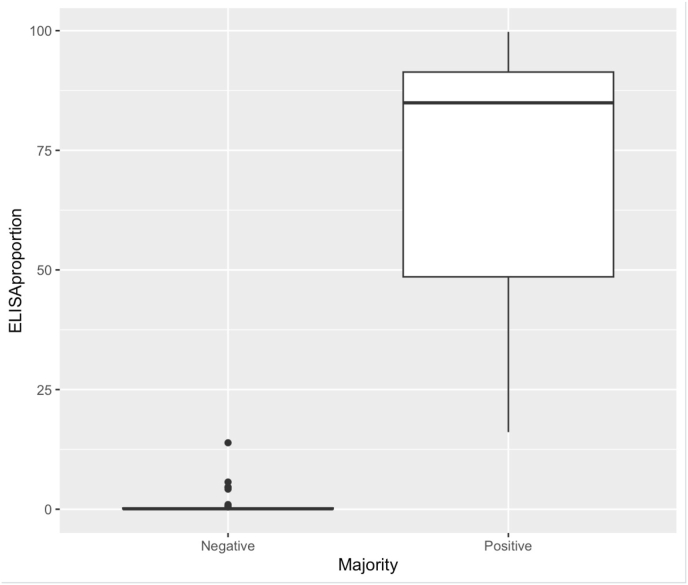
Box plot of *Toxoplasma gondii* serology results from wild boars from Ukraine, obtained using a locally available enzyme-linked immunosorbent assay and majority criteria based on results of three tests (locally available enzyme-linked immunosorbent assay (ELISA), commercial ELISA (ID Screen Toxoplasmosis Indirect Multi-Species), and an indirect immunofluorescence test (IFAT)). ELISA proportion (OD sample/mean OD of positive controls *100) using the locally available ELISA is on the Y-axis and majority criteria is on the X-axis.

Using the majority criteria, 10 of the 92 samples tested with three methods were considered positive, yielding an estimated seropositivity of 10.9% for this subset of samples. An almost perfect agreement was observed between the two ELISAs (Kappa 0.897) (Table 2). The Kappa coefficient of both ELISAs with the majority criteria was 0.946 with a 100% sensitivity (SE) and 98.8% specificity (SP). The IFAT showed Kappa value of 0.508 with a 50% SE and a 96.3% SP. A similar moderate agreement for the Kappa coefficient was obtained when comparing the results of each ELISA with the IFAT. Fig. 2 shows a box plot of the results from the locally available ELISA, by majority criteria using the three tests.

**Table 2:** Agreement between *T*. *gondii* serology screening results obtained using two enzyme-linked immunosorbent assays (ELISA), a locally available ELISA (VectoToxo-antibodies) and a commercial ELISA (ID Screen Toxoplasmosis Indirect Multi-Species).

	Seronegative with the commercial ELISA	Seropositive with the commercial ELISA	Total
Seronegative with the locally available ELISA	80^a^	1	81
Seropositive with the locally available ELISA	1^b^	10	11
Total	81	11	92

a Two samples tested doubtful in the commercial ELISA.

b One sample tested doubtful in the commercial ELISA.

## 4 Discussion

Our study is the first report of *T. gondii* seroprevalence in wild boars from Ukraine. These game animals are important hosts to investigate for *T. gondii*, firstly because their meat is used for human consumption and secondly because they can be considered an indicator host species for presence and spread of *T. gondii*, and the level of environmental contamination with *T. gondii*. Indeed, they belong to the group of recommended animal species for the pre-harvest monitoring (European Food Safety Authority, 2007). Seropositive wild boars were found from several regions, indicating the zoonotic parasite is widespread in Ukraine.

The samples used in this study were collected several years ago, and the epidemiological situation may have changed since. The prevalence of *T. gondii* can change over time (Richomme et al., 2009; Bassi et al., 2021; Kobayashi et al., 2021) depending on a number of factors such as quantity of definitive hosts (Roqueplo et al., 2017; Dubey et al., 1997b; Gauss et al., 2005). In particular, the emergence of ASF in Ukraine has affected the wild boar population (Sytiuk, 2015). Our results can serve as the baseline data before ASF emerged, and further studies could be planned to evaluate the effect of the ASF outbreak on seroprevalence of *T. gondii* in wild boars, as well as the impact caused by the war in Ukraine. Some of the sampled areas have been severely damaged since the start of the war (Clark et al., 2022).

The same locally available ELISA has been previously applied for samples from domestic cats (Galat et al., 2019a) and for samples from horses (Rissanen et al., 2019). For domestic cats, the agreement with a commercial modified direct agglutination test (DAT) was 95.0% and Kappa for dichotomous screening outcome (seronegative or seropositive) was 0.8971, indicating almost perfect agreement (Galat et al., 2019a); whereas for horses, Kappa for dichotomous screening outcome was 0.3838, indicating fair agreement (Rissanen et al., 2019). The results of this study indicate that for wild boar samples, the two ELISAs had moderate agreement with the IFAT. The results may be affected by the cut-offs selected (Basso et al., 2013). Kappa results in our research were lower in comparison with 0.88 agreement between rhoptry-ELISA and IFAT for sera of experimentally infected pigs (Garcia et al., 2006; Machado et al., 2021). The two ELISAs used in this study showed an almost perfect agreement between them.

It is worth noting that the serological testing using the locally available ELISA was performed five years before the samples were tested with the commercial ELISA. This could affect the results, but we did not expect the effect to be major (Dard et al., 2017).

It needs to be emphasized that all three methods are indirect detection methods (Gamble et al., 2005) and measure the humoral reaction of the host to the parasite. Positive results indicate contact or exposure and may not directly reflect whether the host had infective parasites in its tissues (Dubey et al.,1997a; Gardner et al., 2010; Pardini et al., 2012; Puvanesuaran et al., 2013; Casartelli-Alves et al., 2014; Opsteegh et al., 2016; Kunic et al., 2022).

Our overall estimates of seroprevalence are similar to the estimates from e.g. Japan (6.3%), Switzerland (6.7%), and Portugal (7.7%), and lower than those from e.g. Italy (14.0%), Spain (14.7%), Romania (16.0%), Portugal (20.6%), France (23.0%), Estonia (24.0%), the Netherlands (24.4%), Czech Republic (26.2%), Denmark (27.7%), Finland (33.0%), Latvia (33.2%), Poland (37.6% and 37.7%), Slovakia (39.8%), and Slovenia (62.3%) (Bártová et al., 2006; Berger-Schoch et al., 2011; Matsumoto et al., 2011; Opsteegh et al., 2011; Beral et al., 2012; Jokelainen et al., 2012; Deksne and Kirjušina, 2013; Paştiu et al., 2013; Ranucci et al., 2013; Coelho et al., 2014; Calero-Bernal et al., 2015; Jokelainen et al., 2015; Witkowski et al., 2015; Reiterová et al., 2016; Waap et al., 2016; Laforet et al., 2019; Kornacka et al., 2020; Bandelj et al., 2021). It is important to highlight, however, that results from different studies may not be comparable, due to differences in sampling strategies and serological methods used. Importantly, this study showed a wide distribution of seropositive wild boars in Ukraine, and presence of seropositive animals with three serological tests.

The seroprevalence was significantly higher in wild boars over one year of age than those that were younger. This is in line with results from several others studies (Berger-Schoch et al., 2011; Sandfoss et al., 2011; Ranucci et al., 2013; Coelho et al., 2014; Wallander et al., 2015; Opsteegh et al., 2011; Kobayashi et al., 2021; Lizana et al., 2021; Dámek et al., 2023). In other words, older age means longer time to encounter the parasite and become infected. The limited number of wild boars with exact data on their age did not allow to evaluate the effect of age in more detail in this work. According to the results, seroprevalence was higher in the group of animals weighing more than 80 kg, which is in line with results from other studies (Bassi et al., 2021), and could be related to the age of the animals.

More research should be done to study the prevalence of *T. gondii* among definitive and intermediate hosts in Ukraine, as well as the parasite genotyping to be able to compare with other countries. Dissemination the results is important, and not only to scientific audiences (Galat et al., 2018, 2019b, 2023). Public education to prevent toxoplasmosis remains important (Machado et al., 2021), in particular, adequate preparation of meat could help to reduce the risk of human infection (Richomme et al., 2010; Kobayashi et al., 2021).

## 5 Conclusions

Our results indicate that a substantial proportion of wild boars that were hunted in Ukraine had been exposed to the zoonotic parasite *T. gondii*, and the exposure to the parasite appeared widespread in the country. Consuming undercooked wild boar meat should be considered a potential source of *T. gondii* infections of other hosts, including humans. Our results could be used as reference for future studies analyzing effects of other diseases, war and environmental disruption on prevalence and distribution of *T. gondii* in Ukraine.

## Availability of the data

All data generated or analysed during this study are included in this published article.

## Funding

The study was supported by project 0119U100757 funded by the 10.13039/501100007684Ministry of Education and Science of Ukraine, TOXOSOURCES project supported by funding from the European Union's Horizon 2020 Research and Innovation programme under grant agreement No 773830: One Health European Joint Programme, and by the Institute of Parasitology, Vetsuisse Faculty University of Bern, Switzerland.

## Declaration of competing interest

The authors declare that they have no conflict of interest.
